# Carbohydrate Availability and Physical Performance: Physiological Overview and Practical Recommendations

**DOI:** 10.3390/nu11051084

**Published:** 2019-05-16

**Authors:** Fernando Mata, Pedro L. Valenzuela, Jaume Gimenez, Carles Tur, Diogo Ferreria, Raul Domínguez, Antonio Jesús Sanchez-Oliver, José Miguel Martínez Sanz

**Affiliations:** 1 NutriScience, Nutrition & Health Sciences, 14002 Córdoba, Spain; fernando.mata@nutriscience.pt (F.M.); carles.tur@gmail.com (C.T.); df.sportnutrition@gmail.com (D.F.); 2 Systems Biology Department, University of Alcalá, 28801 Madrid, Spain; pedrol.valenzuela@edu.uah.es; 3 Nutrition in Physical Activity and Sports, University of Barcelona, 08007 Barcelona, Spain; jaumegimenez@ub.edu; 4 Faculty of Health Sciences, Universidad Isabel I, 09003 Burgos, Spain; raul.dominguez@ui1.es; 5 Human Motricity and Sports Performance Area, University of Seville, 41004 Seville, Spain; 6 Nursing Department, Faculty of Health Sciences, University of Alicante, San Vicente del Raspeig, 03690 Alicante, Spain; josemiguel.ms@ua.es

**Keywords:** ergogenic aids, exercise, supplement, sport nutrition

## Abstract

Strong evidence during the last few decades has highlighted the importance of nutrition for sport performance, the role of carbohydrates (CHO) being of special interest. Glycogen is currently not only considered an energy substrate but also a regulator of the signaling pathways that regulate exercise-induced adaptations. Thus, low or high CHO availabilities can result in both beneficial or negative results depending on the purpose. On the one hand, the depletion of glycogen levels is a limiting factor of performance during sessions in which high exercise intensities are required; therefore ensuring a high CHO availability before and during exercise is of major importance. A high CHO availability has also been positively related to the exercise-induced adaptations to resistance training. By contrast, a low CHO availability seems to promote endurance-exercise-induced adaptations such as mitochondrial biogenesis and enhanced lipolysis. In the present narrative review, we aim to provide a holistic overview of how CHO availability impacts physical performance as well as to provide practical recommendations on how training and nutrition might be combined to maximize performance. Attending to the existing evidence, no universal recommendations regarding CHO intake can be given to athletes as nutrition should be periodized according to training loads and objectives.

## 1. Introduction

Strong evidence during the last decades has highlighted the importance of nutrition for sport recovery and performance, the role of carbohydrates (CHO) being of special interest [1]. CHO intake is used by athletes to enhance athletic performance via the provision of fuel substrate, to support the immune system, and to improve the bioavailability of other supplements (e.g., protein supplements) [2].

When exercising at high intensities, CHO become the main energy substrate, and thus glycogen depletion is considered an important limiting factor of performance [3]. A high exogenous CHO availability during exercise (78–90 g/h, approximately) [4,5] facilitates performance through the sparing of hepatic glycogen, avoiding potential hypoglycemia, as well as through the maintenance of a high CHO oxidation rate, which enables a higher exercise intensity [6]. For this reason, in order to attain a high performance level during exercises that rely on the glycolytic pathway (be they resistance or endurance exercises), it would be recommendable to start exercising with full glycogen stores as well as to supply CHO during the effort.

The nutritional strategies needed to meet daily CHO requirements have been discussed in recent years with recommendations being made attending to different variables, such as the training/competition phases, the number of training sessions, and the periodization of the training [7]. The aim of this review is to provide a holistic overview of CHO periodization and its effects on sports performance.

## 2. Carbohydrates and Training Adaptations

The adaptive response to training is determined by the type of exercise performed and by the training load applied (e.g., duration, intensity frequency) [8] but also by other factors such as nutritional status [9,10]. Thus, nutritional intake can modulate exercise-induced adaptations. 

The availability of energy during exercise modulates the physiological response to training [11]. Nowadays, glycogen is not only considered as an energy substrate but also as a regulator of the signaling pathways that regulate the adaptations in response to exercise [12,13]. Thus, manipulating the endogenous (glycogen stores) and exogenous (CHO intake) availability of CHO before and/or after training sessions may be used to alter exercise-induced adaptations [14].

### 2.1. Carbohydrates Availability and Resistance Training-Induced Adaptations

It has been observed that the anabolic response after resistance training depends on the status of glycogen stores. This anabolic response is regulated by complex signaling pathways, one of the most important being the mTOR pathway [15]. Glycogen availability exerts a negative regulatory activity over AMPK. Thus, in situations of energy deficit (low glycogen availability) the AMPK pathway, which is involved in the generation of ATP, is activated, whereas the mTOR pathway, which requires energy for its activity, is inhibited [16,17]. Although the evidence is not unanimous, low levels of glycogen could inhibit the hypertrophic response to resistance training [18,19] and increase proteolysis [20,21]. For this reason, it would be convenient to start resistance exercises with replenished glycogen stores [16,17].

The hypertrophic adaptations to resistance exercises could be also facilitated by an increase in the post-exercise hormonal response. Insulin is an anticatabolic, which achieves muscle protein anabolism by the inhibition of muscle protein breakdown [22], so CHO intake after exercise could be beneficial to potentiate exercise-induced adaptations. However, evidence is mixed in this regard, and it seems that supplying an optimal amount of high-quality proteins, specifically proteins that contain an adequate dose of leucine (e.g., 3–4 g/dose) [23] after exercise can be enough to achieve a positive protein balance and stimulate an anabolic response [24]

In addition to allowing better rates of work, another goal of resistance training programs consists of maintaining or stimulating a positive nitrogen balance [25]. Thus, CHO ingestion maintains higher levels of insulin concentration, which is a hormonal response that stimulates the uptake and storage of CHO in tissues and decreases the use of amino acids [26]. Therefore, CHO ingestion during the effort could be useful to reduce amino acid degradation, favoring a positive nitrogen balance.

### 2.2. Carbohydrates Availability and Endurance Training-Induced Adaptations

Classical studies have already demonstrated the beneficial role of a high-CHO diet for endurance exercise performance [27,28]. A high CHO availability is beneficial in endurance exercise during competitions or in training sessions in which a high level of intensity is required [8,29]. Thus, a high CHO intake (10 g/kg) has been recommended the day before a competition (more precisely ~36 h from the last training session) and physical inactivity to ensure glycogen supercompensation [1,9]. However, it may be unnecessary in sessions where exercise intensity is not important [1]. According to the last position of the American College of Sport Medicine, nutritional recommendations have traditionally focused on optimal nutrition during competitions, but a universal recommendation of an optimal intake of CHO might not be appropriate as there is a great diversity of goals and training loads across training microcycles and macrocycles [1]. 

The acute and chronic adaptations to endurance exercise have been widely described, including mitochondrial biogenesis, a shift in the fiber-type prevalence toward a slower-twitch phenotype, improved-oxidative metabolism, and increased-capillary density [16,18,30]. Several studies demonstrated that during periods in which CHO availability was strategically reduced (known as “training low”) there was an increase of molecular markers related to endurance training-induced adaptation [31,32]. Considering these responses, it could be important to differentiate between the optimal nutrition to compete and to train [32]. Thus, whereas during high-intensity sessions or competitions it is essential to train with a high CHO availability, it might be beneficial to exercise with a reduced CHO availability in low-intensity ones [33]. This paradigm is known as “train low, compete high” and could be applied specifically when exercise intensity is close to or below the first ventilatory threshold (VT1) as this intensity matches with the maximum recruitment of slow-twitch motor units [34].

The AMPK-PGC-1α signaling pathway plays a key role in the adaptations to endurance exercise through inducing mitochondrial biogenesis and improving oxidative capacity [35,36]. A decrease in glycogen content and an increase in the AMP:ATP ratio are two important signals that trigger the activation of the AMPK pathway [31,37]. The adaptive response to endurance exercise with a low glycogen availability is regulated by both AMPK and p38MAPK, converging both pathways in the regulation of key transcriptional factors and co-activators, such as PGC-1α, p53, and PPARδ [37,38]. Although AMPK and p38MAPK, and subsequently PGC-1α, are activated by endurance exercise per se, these molecular signals seem to be amplified when glycogen stores are low [31].

The first study analyzing the adaptations to exercising with a low CHO availability possibly took place in 1983, when explorers in the Arctic and Antarctica seemed to adapt to these kind of diets [39]. Nowadays, several studies have showed how performing endurance exercise with a low CHO availability induced greater physiological adaptations than performing the same training session with a normal CHO availability [31,40]. For instance, “training low” augmented the expression of genes related to mitochondrial biogenesis and oxidative metabolism [41,42,43]. Moreover, a reduction of CHO availability during 3–10 weeks improved enzymatic activity, increased the content of proteins involved in oxidative metabolism, and increased the capacity to oxidize lipids in endurance athletes [44,45,46].

Despite the aforementioned physiological adaptations, controversy exists regarding the effectiveness of periodized nutritional strategies for the improvement of exercise performance with some studies reporting beneficial effects [32,38,47,48,49] and others showing no benefits or even performance impairments [44,45,46,50,51]. Burke et al. recently reported that elite race walkers that had followed a very low-CHO diet during 3 weeks increased their fat oxidation rate and decreased their body mass, which could be considered a beneficial physiological adaptation [50]. However, their performance was impaired in comparison with diets supplying a higher amount of CHO [50]. Similarly, another study analyzed the effects of CHO restriction during four weeks in elite endurance athletes and did not find beneficial effects on their performance [51]. By contrast, Marquet et al. performed a study in which athletes altered their CHO intakes attending to the intensity of their training sessions, reducing glycogen availability during low-intensity training sessions (which were performed the day after a high-intensity training session with no posterior CHO reposition) and increasing glycogen availability (i.e., increased CHO intake) when higher intensities were required. The authors observed that this strategy yielded performance benefits (improved 10-km running performance, improved cycling efficiency, and increased time to exhaustion at 150% of the maximal aerobic power in a cycle ergometer) compared to a group that consumed an even distribution of CHO during all training sessions. An important aspect of this study was that the total CHO intake was the same in both experimental groups, and they just differed in the timing of this intake. These results suggested that the timing of a CHO intake should be individualized and periodized every day depending on the intensity of the training session [38]. Thus, nutritional periodization might be beneficial for athletes’ performance [1,10].

### 2.3. Protocols for Achieving a Low CHO Availability

Different protocols for the manipulation of CHO availability have been studied, notably training twice on the same day [46,49,52], exercising in a state of fasting [41], or restricting CHO intake during [44] or after exercise [41]. Another strategy that is growing in popularity is ‘sleeping low’, which consists of performing a high-intensity training session in an afternoon/night with a high CHO availability followed by a period of CHO restriction during that night and a low-intensity training session while fasting on the following day [38]. This strategy has been reported to enhance exercise-induced adaptations and performance in endurance athletes [38], and neither the immune system nor the exercise intensity during the high-intensity training session seem to have been affected [53].

Despite the theoretical basis supporting ‘training low’ strategies, there are some potential risks associated with the long-term maintenance of a low CHO availability, including an impairment of the immune system, a reduced CHO oxidation rate during exercise—and consequently an inability to exercise at high intensity, and an increased oxidation of muscle proteins [37]. These limitations can be even more exacerbated if athletes and coaches incorrectly interpret the term “training low” as “training zero”, which means training chronically with an extreme CHO restriction [31]. In order to avoid the deleterious effects of energy deficits on protein synthesis, consuming a leucine-rich protein close to the time of the training session has been proposed [32]. Some strategies can also be used to avoid lowering training intensity when exercising with a low CHO availability. For instance, studies have suggested that caffeine intake, mouth rinsing with a CHO solution during exercise, or even mouth rinsing with a caffeinated solution can attenuate the reduction of training intensity in these situations [54,55].

## 3. Carbohydrates and Supplementation Timing in Exercise

### 3.1. Carbohydrates Supplementation before Exercise

It has been recommended that athletes should take 1–4 g/kg of CHO 1–4 h before exercise, but an excessive intake might also adversely affect performance [30]. This is usually caused because the metabolic effects of the insulin response (a reduction in fat mobilization and an increase in CHO use) can promote premature fatigue (reactive or rebound hypoglycemia). Some athletes may develop symptoms similar to those of hypoglycemia, but they are not always related to low glucose concentrations in blood [4]. The strategy suggested to decrease this problem is ensuring at least 1 g/kg of CHO in a pre-event meal with a protein source, some high-intensity efforts in a pre-exercise warmup, and consuming CHO during exercise. Few studies have found adverse effects on performance, and most studies have reported no change or an improvement in performance; thus, to avoid CHO intake in the hour before exercise is an unfounded myth [56,57,58].

Research examining the effects of different levels of CHO restriction has produced contradictory results. Cholewa et al. recently concluded that a low glycogen or CHO availability does not negatively affect acute resistance-exercise performance when the training volume is lower than ~8 sets with a duration <45 min (low volume and high intensity), but increasing the CHO intake after a restriction period may enhance acute strength performance, muscular endurance, and hypertrophy [59]. They also suggested that acutely increasing blood glucose before resistance exercise might improve performance in training sessions >50 min (high volume and moderate intensity) [53]. Getzin and colleagues indicated that runners and triathletes with higher blood glucose levels post-competition (CHO intake before and during) tended to perform well and fare better upon finishing [60]. For these reasons, athletes should be encouraged to increase CHO intake in their diets in order to increase their muscle glycogen stores before a competition.

### 3.2. Carbohydrate Supplementation during Exercise

The role of CHO supplementation during endurance exercise is well-established in scientific literature [6,61]. For instance, a recent systematic review showed that 82% of the included studies reported significant benefits of CHO supplementation on endurance performance [62].

Although CHO supplementation is overall recommendable during exercise, it is important to remark upon some considerations [63]. Not all CHO sources supply energy or are digested and absorbed at the same rates. The maximum rate of a single CHO source’s absorption is 60 g/h, corresponding to CHO, such as glucose, sucrose, maltose, or maltodextrin [64]. Other CHO sources such as fructose and galactose present lower absorption rates [65]. If supplied in excessive amounts (i.e., in excess of the capacity for intestinal absorption), CHO intake can lead to gastrointestinal discomfort [66]. During long-duration endurance exercise, it may be beneficial to supply a higher amount of CHO [67]. For this purpose and considering the aforementioned limit of absorption (60 g/h), supplying CHO sources that use a different transporter to enter the luminal membrane of the intestinal wall has been recommended. Thus, supplying 60 g/h of glucose and an additional amount of fructose, which is possible as both CHO use different transporters (SGLT1 and GLUT5, respectively), would increase the CHO oxidation rate [68], reduce gastrointestinal problems [10], and thus benefit performance in comparison with the intake of only one type of CHO [69]. Thus, the CHO absorption rate could become 90 g/h, approximately [4].

The ergogenic properties of CHO might not only be due to metabolic mechanisms but also to central ones, an increased performance during exercise of less than 1 h concomitant with changes at the brain level after swallowing (without ingesting it) a CHO solution having been reported [70,71].

Resistance exercise also exerts a glycogenolytic effect [72]. For instance, a single resistance training session can result in reductions in muscle glycogen stores of as much as 24–40% [73], which may impair performance [74]. It has been shown that CHO supplementation during resistance exercise attenuates glycogen depletion [75], which suggests that it could be beneficial to improve performance during this type of exercise [76]. However, whereas some studies support the ergogenic properties of CHO when supplied before or during resistance exercise [77,78], others have failed to find these benefits [75,79,80]. It could be hypothesized that CHO supplementation would provide more benefits during training sessions in which glycogen stores are depleted at a higher rate (i.e., high-volume resistance training sessions).

### 3.3. Post-Exercise Glycogen Restoration

As glycogen depletion is a limiting factor of performance, it is important to start competitions or those training sessions in which a high level of performance is required with glycogen stores as full as possible [81]. Glycogen depletion stimulates the enzyme responsible for its synthesis (glycogen synthase) and increases insulin sensitivity and membrane permeability to glucose [82,83], facilitating glycogen re-synthesis. A classic study that popularized the term “metabolic window of opportunity” found that the ratio of glycogen re-synthesis was much higher if CHO intake took place within 2 h post-exercise than if CHO were supplied later [84], which is of special importance if little recovery time is available between two training sessions or competitions (e.g., two training sessions on the same day) since the rate of glycogen re-synthesis is only about 5% per hour [81]. Nevertheless, in the later phase of recovery (4–24 h), supplying an optimal amount of CHO (1.2 g/kg/h, approximately) seemed to be more important than the timing of ingestion [81]. Moreover, glycogen re-synthesis may be favored by those elements that increase gastric emptying, insulin response and cell osmolarity. Thus, CHO sources with rapid absorption rates or a high-glycemic index (e.g., glucose) could facilitate glycogen re-synthesis more than other CHO sources (e.g., fructose), and its combination with protein, which increases the insulin response, or the addition of creatine, which increases cell osmolarity, could maximize the response [81]. The combination of glucose with fructose ingestion during recovery has recently been highlighted as an effective strategy to (1) lower gastrointestinal distress and (2) accelerate liver glycogen resynthesis (due to the faster intestinal absorption of glucose-fructose mixtures when compared to the ingestion of either glucose or fructose in isolation) [85]. Moreover, the co-ingestion of carbohydrates with caffeine immediately after exercise has also been reported to enhance glycogen re-synthesis [56,86] while the doses of caffeine could affect the quantity and quality of sleep.

## 4. Conclusions and Practical Applications

When exercising at moderate or high intensity, CHO are the main energy substrate, and glycogen depletion is a major limiting factor of performance. If the goal is to achieve performance during that training session or competition, it is convenient to supply an optimal amount of CHO before and during exercise. For this purpose, it is necessary to take 1–4 g/kg of CHO (low-glycemic index) 1–4 h before exercise and a maximum of 90 g/h of CHO (a 2:1 relation between glucose and fructose). In addition, for optimal glycogen replenishment, it is necessary to take different intakes of high-glycemic index CHO (1 g/kg/h) in the 2–4 h after the training session. For this purpose, it would also be recommendable to increase the daily intake of CHO (10 g/kg) the day (24–36 h) before a competition, whenever possible.

Sometimes, training with a low CHO availability during endurance exercise can be useful in those periods of the season when the purpose is to maximize adaptations such as mitochondrial biogenesis and improved fatty acid oxidation. Because maintaining these strategies chronically (>3 weeks) could result in an impairment of the immune system, sleep quality, and protein balance, nutritional periodization might be an effective strategy to maintain metabolic flexibility and exercise intensity. Thus, whereas high intensity sessions can be performed with a high CHO availability, CHO intake can be restricted in those training sessions in which exercise intensity is not compromised by a low CHO availability, such as those below the first ventilatory threshold (see Table 1).

## Figures and Tables

**Table 1:** An example of a CHO periodization during a week for an endurance athlete.

	Monday	Tuesday	Wednesday	Thursday	Friday	Saturday	Sunday
Morning	1.5 g/kg	LIT	1.5 g/kg	LIT	1.5 g/kg	LIT	1.5 g/kg
		2 g/kg		2 g/kg		1.5 g/kg	
Afternoon	2 g/kg	2 g/kg	2 g/kg	2 g/kg	2 g/kg	2 g/kg	1.5 g/kg
Evening	1.5 g/kg	1 g/kg	1.5 g/kg	1 g/kg	1.5 g/kg	1 g/kg	1.5 g/kg
	HIT *		HIT *		HIT *		
Dinner	<0.5 g/kg	2 g/kg	<0.5 g/kg	2 g/kg	<0.5 g/kg	1.5 g/kg	1.5 g/kg

HIT: high intensity training; LIT: low intensity training (<ventilatory threshold); * During HIT, it is necessary to intake 60–90 g/h (2:1 glucose:fructose) of supplement foods and liquids.
